# Epigenetic silencing of DSC3 is a common event in human breast cancer

**DOI:** 10.1186/bcr1273

**Published:** 2005-06-16

**Authors:** Marc M Oshiro, Christina J Kim, Ryan J Wozniak, Damian J Junk, José L Muñoz-Rodríguez, Jeanne A Burr, Matthew Fitzgerald, Sangita C Pawar, Anne E Cress, Frederick E Domann, Bernard W Futscher

**Affiliations:** 1 Departments of Pharmacology and Toxicology, Arizona Cancer Center, University of Arizona, Tucson, AZ, USA; 2 Department of Surgery, Arizona Cancer Center, University of Arizona, Tucson, AZ, USA; 3 Department of Cell Biology and Anatomy, Arizona Cancer Center, University of Arizona, Tucson, AZ, USA; 4 Department of Radiation Oncology, Free Radical and Radiation Biology Program, Holden Comprehensive Cancer Center, University of Iowa, Iowa City, IA, USA

## Abstract

**Introduction:**

Desmocollin 3 (DSC3) is a member of the cadherin superfamily of calcium-dependent cell adhesion molecules and a principle component of desmosomes. Desmosomal proteins such as DSC3 are integral to the maintenance of tissue architecture and the loss of these components leads to a lack of adhesion and a gain of cellular mobility. *DSC3 *expression is down-regulated in breast cancer cell lines and primary breast tumors; however, the loss of *DSC3 *is not due to gene deletion or gross rearrangement of the gene. In this study, we examined the prevalence of epigenetic silencing of *DSC3 *gene expression in primary breast tumor specimens.

**Methods:**

We used bisulfite genomic sequencing to analyze the methylation state of the *DSC3 *promoter region from 32 primary breast tumor specimens. We also used a quantitative real-time RT-PCR approach, and analyzed all breast tumor specimens for *DSC3 *expression. Finally, in addition to bisulfite sequencing and RT-PCR, we used an *in vivo *nuclease accessibility assay to determine the chromatin architecture of the CpG island region from *DSC3*-negative breast cancer cells lines.

**Results:**

*DSC3 *expression was downregulated in 23 of 32 (72%) breast cancer specimens comprising: 22 invasive ductal carcinomas, 7 invasive lobular breast carcinomas, 2 invasive ductal carcinomas that metastasized to the lymph node, and a mucoid ductal carcinoma. Of the 23 specimens showing a loss of *DSC3 *expression, 13 (56%) were associated with cytosine hypermethylation of the promoter region. Furthermore, *DSC3 *expression is limited to cells of epithelial origin and its expression of mRNA and protein is lost in a high proportion of breast tumor cell lines (79%). Lastly, DNA hypermethylation of the DSC3 promoter is highly correlated with a closed chromatin structure.

**Conclusion:**

These results indicate that the loss of *DSC3 *expression is a common event in primary breast tumor specimens, and that *DSC3 *gene silencing in breast tumors is frequently linked to aberrant cytosine methylation and concomitant changes in chromatin structure.

## Introduction

Aberrant cytosine methylation of CpG dinucleotides in the promoter region of genes is often associated with changes in their chromatin structure and transcriptional silencing of the gene during carcinogenesis and tumor progression [1-7]. Cytosine methylation has been shown to play a fundamental role in breast tumor progression, as silenced genes have been identified that fall into each of the six 'acquired capabilities of cancer' as described by Hanahan and Weinberg [8]. Targeted genes include regulators of cell cycle, maintainers of genomic integrity, tumor suppressors, as well as adhesion molecules [9]. Examples of hypermethylated genes in breast cancer include: maspin, E-cadherin, BRCA1, ras association domain family 1A, tissue inhibitor of metalloproteinase-3, and A Disintegrin And Metalloprotease domain 23 gene [1,10-17].

Desmosomes, together with adherens junctions, represent the major adhesive cell-junctions of epithelial cells [18-20]. E-cadherin is one example of an integral component of adherens junctions whose role in breast tumor progression has been clearly established [10,13,21]. The participation of desmosomal components in cancer, however, is enigmatic. Desmosomes are multifaceted intracellular junctions that participate in cell adhesion and maintenance of normal tissue structure in the epidermis [20,22]. Desmocollins (DSCs) are members of the cadherin superfamily, and fundamental members of the desmosome. DSC family members are uniquely expressed in epidermal tissue, with DSC2 being expressed in all desmosome-bearing tissues, while DSC1 and DSC3 expression is restricted to certain specialized epithelia, mainly stratified squamous epithelia [23,24]. Furthermore, DSC1 is expressed in the higher terminally differentiated cell layers, while DSC3 is mainly expressed in the basal layers [23,24]. In addition, the DSCs are present in both 'a' and 'b' isoforms, resulting from the alternate splicing of exon 16 [25]. They differ with respect to their carboxy-terminal end, with the 'b' form having a shortened carboxy-terminal domain that removes the major binding site for plakoglobin [26].

One of the more intriguing functions of desmosomal proteins as they relate to cancer is their ability to inhibit cell motility. Notably, Tselepis *et al*. [27] showed that the expression of multiple desmosomal components (DSC, desmoglein, and plakoglobin) were sufficient to induce adherence of the normally non-adherent invasive L929 fibroblast. This induced adhesion could be blocked by the addition of short peptides corresponding to the putative cell adhesion recognition sites of DSC and desmoglein. In addition, the introduction of these desmosomal proteins was also sufficient to inhibit L929 invasion into collagen gels. Recently, functional studies that targeted the inhibition of *DSC3 *with dominant negative constructs showed that DSC3 expression is required for the formation of desmosomes and adherens junctions [28]. In total, these studies support the idea that intact desmosomes can inhibit cellular motility.

Down-regulation of *DSC3 *in breast cancer was first reported by Klus [29]. They showed that *DSC3 *was expressed in normal breast while its expression was down-regulated in both primary breast tumors and breast tumor cell lines. We recently performed two-color fluorescence cDNA microarray experiments to identify p53 response genes in human breast tumor cell lines [6]. Our results identified *DSC3 *as a p53 response gene whose expression was downregulated in 80% of breast tumor cell lines tested. In addition, analysis of breast cancer cell lines showed that *DSC3 *is silenced in association with cytosine hypermethylation and histone deacetylation [6]. Therefore, the loss of *DSC3 *expression in the cell lines appears to be due to both epigenetic and genetic changes.

In this study, we extend our *in vitro *analysis of *DSC3 *to the investigation of the frequency of epigenetic silencing of *DSC3 *expression in primary breast tumor specimens. DNA from freshly isolated tumor specimens was analyzed for cytosine methylation by sodium bisulfite sequence analysis while RNA from the same tumors was analyzed by quantitative real-time RT-PCR for *DSC3 *expression. Our results show that epigenetic silencing of *DSC3 *is a common event in primary breast tumor specimens, as 72% of breast carcinomas analyzed showed a loss of *DSC3 *expression and that the loss of expression strongly correlated with cytosine methylation of its promoter region in 56% of *DSC3*-negative breast carcinomas analyzed, and 41% of all specimens analyzed.

Concurrently, we analyzed a panel of breast tumor cell lines for *DSC3 *expression and concomitant cytosine methylation of its promoter region. As aberrant cytosine methylation of promoter regions is associated with alterations to chromatin structure, we also compared the *in vivo *nuclease accessibility of the *DSC3 *promoter region in normal and tumor breast cell lines. Our results indicate that the loss of DSC3 is a common event in breast tumor cell lines at both the mRNA and protein levels and that the loss of expression is frequently correlated with cytosine methylation of its promoter region and an inaccessible chromatin structure. These results indicate that epigenetic silencing of *DSC3 *is an underlying event in breast tumorigenesis.

## Materials and methods

### Cell culture and manipulations

Normal human mammary epithelial cells (HMECs) and human prostate epithelial cells were obtained from Clonetics (San Diego, CA, USA), fetal skin keratinocytes from Cell Applications (San Diego, CA, USA); these were grown according to manufacturers' instructions. Human foreskin fibroblasts were maintained and cultured in the Arizona Cancer Center Cell Culture Shared Service (Tucson, AZ, USA). Peripheral blood lymphocytes were obtained from the whole blood of healthy donors in accordance with the health insurance portability and accountability act of 1996 (HIPAA) guidelines. Briefly, whole blood was collected into BD Vacutainer CPT cell preparation tubes containing sodium heparin (Becton Dickinson, Franklin Lakes, NJ, USA) and processed according to the manufacturer's protocol. Primary cultures of normal human oral keratinocytes were established and maintained in short-term culture as described [30-32]. Primary cultures of human airway epithelial cells were obtained by enzymatic digestion of bronchial samples from lung transplants and maintained in short-term culture as described [33,34].

The MCF10A, MDA-MB-453, MDA-MB-435, MDA-MB-231, MDA-MB-157, MDA-MB-468, BT549, ZR-75-1, and HS578T breast cancer cells were obtained from the American Type Culture Collection (Rockville, MD, USA). The HaCaT cells, a normal immortalized keratinocyte cell line [35], were obtained from Norbert E Fusenig (German Cancer Research Center, University of Heidelberg, Heidelberg, Germany). The early passage sporadic breast cancer cell lines UACC1179, UACC2087, UACC893, UACC3133, UACC3199, and UACC2648 were developed and maintained at the Arizona Cancer Center Cell Culture Shared Service.

### Breast tumor specimens

Thirty flash frozen breast cancer tissue specimens were obtained from patients who underwent surgery for breast cancer, either lumpectomy or mastectomy, at the University Medical Center in Tucson, AZ, from 2003 to 2004. All patients signed surgical and clinical research consents for tissue collection in accordance with the University of Arizona Institutional Review Board and HIPAA regulations. At the time of surgery, a 1–3 cm section of the tumor was immediately snap frozen in liquid nitrogen and stored in our prospective breast tissue bank at -80°C. From each tissue block, a series of 5 micron sections were cut and stained with hematoxylin and eosin (H&E) for pathological evaluation. All of the H&E slides were reviewed by one breast pathologist to determine the integrity of the tumor specimen and this was correlated with the clinical pathologic review performed by an independent pathologist.

### Nucleic acid isolation

Total RNA was isolated from cells using an RNeasy^® ^Mini or Midi Kit (Qiagen, Valencia, CA, USA), and genomic DNA was isolated using the QIAamp DNA Mini Kit (Qiagen, Valencia, CA). Isolation of RNA from frozen breast tumor specimens was done as follows: 30–50 μg of tissue was disrupted in a 1.5 ml RNAase free tube with an RNAase free Pellet Pestle (Kimble-Kontes, Vineland, New Jersey, USA) then passed through a 21 gauge needle to homogenize the sample. Following homogenization, RNA was isolated using the RNeasy^® ^Mini kit. Isolation of DNA from frozen breast tumor specimens was done using a Medimachine (BD Biosciences, San Jose, CA, USA). Briefly, a 50 μm Medicon (BD Biosciences) was washed twice with 1 ml of TKM1-NP buffer (10 mM Tris-HCl, pH 7.6, 10 mM KCl, 10 mM MgCl2, 2 mM EDTA, and 2.5 μl/ml NP40) then a 30–50 μg piece of frozen breast tumor tissue was further cut into 3–6 mm^3 ^pieces then placed into the Medicon filled with 1 ml of TKM1-NP buffer. The tissue was disaggregated for 30 s then allowed to rest for 20 s and then disaggregated for another 30 s. The cell suspension was then passed through 100 μm Filcon (BD Biosciences) into a 15 ml conical tube. Disaggregation in the Medicon was repeated four to six more times to completely disaggregate the tissue. Once done, the cell suspension was spun down for 10 minutes at 250 × g at 4°C. Completion of DNA isolation was done using the Qiagen DNA mini kit, Tissue Protocol. RNA and DNA samples were quantified by UV absorbance measurements at 260 nm. Furthermore, all breast tumor specimen RNAs were run out on an Agilent RNA Labchip (Agilent Technologies, Waldbronn, Germany) for quantitative and qualitative assessment of the RNA.

### Sodium bisulfite genomic sequencing of the DSC3 promoter

Genomic DNA (5 μg) was modified with sodium bisulfite under conditions previously described [1]. The *DSC3 *promoter was amplified from the bisulfite-modified DNA by two rounds of PCR using nested primers specific to the bisulfite-modified sequence of the *DSC3 *CpG Island. First round primers were: UTDSC3_F1, GATTGGGGTTTTGTATTGAGA; UTDSC3_R1, TTAACCTCTCTCAAACTTACC. Second round primers were: UTDSC3_F2, ATTTGGGTTGTTAGGGTTTTTTT; UTDSC3_R2, AAAACAACTTCACTTCTAAAACC. Both rounds of PCR were performed under the same parameters, with 1% of the first round PCR product serving as the template in the second round of PCR. PCR amplification was performed under the following conditions: 94°C for 4 minutes followed by 5 cycles of 94°C for 1 min, 56°C for 2 min, 72°C for 3 min, then 35 cycles of 94°C for 30 s, 56°C for 2 min, 72°C for 1.5 min, and ending with a final extension of 72°C for 6 min.

The resultant PCR product was cloned into a TA vector according to the manufacturer's instructions (pGEM-T-Easy cloning kit; Promega, Madison, WI, USA)). Ten positive recombinants were isolated using a Qiaprep Spin Plasmid Miniprep kit (Qiagen) according to the manufacturer's instructions and sequenced on an ABI automated DNA sequencer (Applied Biosystems, Foster City, CA, USA). The methylation status of individual CpG sites was determined by comparison of the sequence obtained with the known *DSC3 *sequence. The number of methylated CpGs at a specific site was divided by the number of clones analyzed (minimum of 10 in all cases) to yield a percent methylation for each site.

### Western blot

Cells were lysed by incubating on ice for 2 minutes in RIPA buffer (1 × PBS containing 1% NP40, 0.5% deoxycholate, and 0.1% SDS) with 1 mM phenylmethylsulfonyl fluoride (Boehringer Mannheim Corp, Indianapolis, IN, USA) added directly before use. Samples were sonicated using five pulses of 1 s each. Protein concentration was determined by BCA (bicinchoninic acid) assay (Pierce, Rockford, IL, USA). Whole cell lysates of 20 μg of total protein were diluted in 2 × non-reducing sample buffer and then boiled for 3 minutes before loading onto a 7.5% polyacrylamide gel for analysis. Proteins resolved in the gel were electrotransferred to Millipore Immobilon-P PVDF membrane (Millipore, Bedford, MA, USA). The membranes were blocked in 5% non-fat milk in TBST (10 mM Tris-HCl, pH 7.5, 150 mM NaCl, 0.1% Tween 20). Primary mouse monoclonal antibody anti-Desmocollin Clone Dsc3-U114 (Research Diagnostics Inc., Flanders, NJ, USA) was diluted 1:10 in 5% non-fat milk/TBST and incubated with the membrane overnight at 4°C. Membranes were washed three times with TBST, incubated with a donkey anti-mouse horseradish peroxidase-conjugated secondary antibody (Chemicon International, Temecula, CA, USA), washed six more times with TBST, visualized with an ECL Western Blotting Detection Kit (Amersham Biosciences, Piscataway, NJ USA), and detected with BioMax MR film (Kodak, Rochester, NY, USA).

### Quantitative real time RT-PCR

For real time quantitative RT-PCR analysis of *DSC3 *and *GAPDH *gene expression, a reverse transcription step was performed using TaqMan^® ^Reverse Transcription Reagents (Roche Molecular Systems, Branchburg, NJ, USA) and 250 ng of total RNA in a 50 μl reaction. The reverse transcription reaction was primed with random hexamers and incubated at 25°C for 10 minutes followed by 48°C for 30 minutes, 95°C for 5 minutes and a chill at 4°C. For the PCR reaction, 10 ng of cDNA was used in accordance with the protocol outlined in the ABI user manual (Applied Biosystems). *DSC3 *and *GAPDH *primer probes were purchased from ABI Assays-on-demand (Fwd Primer, CCAATCCGGTTTCAGAAGTGA; Rev Primer, CTCGCCGCTGCTTGTTTT; FAM Probe, CTCTCTCAGGCTTGCC) were used and data collected using the ABI Prism 7000 real-time sequence detection system (Applied Biosystems). Differences in expression were determined using the comparative Ct method described in the ABI user manual (Applied Biosystems).

### Chromatin accessibility assays

Chromatin accessibility assays were performed as previously described (Oshiro *et al*. [6]) with minor modifications. Ten million cells were washed twice with ice cold 1 × PBS, gently scraped and collected by centrifugation. Nuclei were extracted by resuspension of cells in ice cold 1 × RSB (10 mM Tris HCl, pH 8, 3 mM MgCl_2_, 10 mM NaCl, 0.05% NP40). The nuclei were collected by centrifugation, resuspended in appropriate 1 × restriction endonuclease buffer, and divided into two aliquots of 200 μl/aliquot. MspI (0 or 75 units; Gibco BRL, Bethesda, MD, USA) was added to the nuclei and incubated at 37°C for 15 minutes. Genomic DNA was isolated using the QIAamp DNA Mini Kit (Qiagen) and ligated to linkers specific for the *MspI *ends. The linker 'marks' accessible sites of chromatin, and acts as the primer sequence for PCR along with the *DSC3 *promoter specific primer. Primers were: linker specific primer, GGATTTGCTGGTGCAGTACT; first round gene specific primer, CCTAAATCCCTTTTCAAGTCT; second round gene specific primer, CTCAAAACAAAAAGCTCAGTCCAGA. To increase specific amplification of our band of interest, a second round of PCR was performed using a 1:1000 dilution of the first round PCR product, adding a second, nested primer that was specific for the genomic region being analyzed and internal to the first region-specific primer.

First round PCR was performed using RTG PCR beads (Pharmacia, Piscataway, NJ, USA) to amplify 100 ng of linkered DNA. The initial step in the first round PCR reaction was a 15 minute incubation at 72°C followed by a denaturation at 95°C for 2 minutes then 25 cycles of 95°C for 30 s, 55°C for 1 min, 72°C for 2 s and a final extension at 72°C for 5 minutes. The second round of PCR was performed using the ABI Prism 7000 real-time sequence detection system (Applied Biosystems). For the nested PCR step, 25 pmol (1 μl) of internal *DSC3 *specific primer was added to 5 μl of diluted first-round product (1:1000), 19 μl of PCR water and 25 μl of 2 × SYBR^® ^Green PCR Master Mix (Applied Biosystems). The PCR conditions for this second round of PCR were as follows: a 10 minute denaturation at 95°C and 40 cycles of 94°C for 1 min, 56°C for 40 s and 72°C for 30 s. Relative levels of chromatin accessibility were determined using the comparative Ct method. Real-time PCR products were also separated on a 3% TBE agarose gel to verify the presence of a single PCR product of the appropriate size (241 bp).

## Results

**Figure 1:**
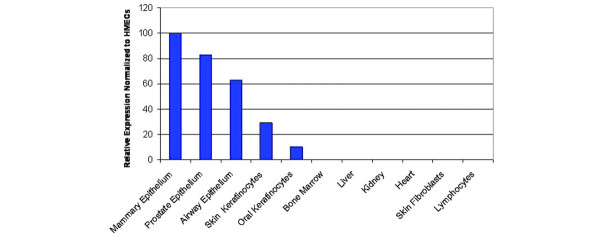
*DSC3 *expression is restricted to a subset of normal human epithelial cell types. *DSC3 *expression relative to human mammary epithelium cells (HMECs) was assessed by real-time quantitative RT-PCR; *GAPDH *expression was used to normalize the data.

Relative levels of *DSC3 *mRNA expression from a panel of normal tissue RNA were determined by quantitative real time RT-PCR analysis. *DSC3 *mRNA levels were normalized to the ubiquitously expressed *GAPDH *gene, then expression values reported relative to the primary HMEC line expression (Fig. 1). *DSC*3 expression was limited to certain epithelial cell types, including those of the airway, breast, skin, prostate, and mouth. *DSC3 *was undetectable in the following non-epithelial cell types: skin fibroblasts, lymphocytes, bone marrow, heart, and kidney. Therefore, expression analysis of *DSC3 *shows a significant cell type specific pattern of expression, which is limited to cells of epithelial origin, including breast epithelium.

To confirm and extend previous studies [6,29], we analyzed 32 frozen breast cancer specimens from patients who underwent lumpectomy or mastectomy randomly obtained from patients who underwent surgery at the University Medical Center in Tucson, AZ. Our data set comprised 32 specimens: 24 invasive ductal carcinomas (IDC), two of which are metastatic IDCs isolated from patients' lymph nodes, seven invasive lobular carcinomas (ILCs), and one mucoid ductal carcinoma. Incidentally, we received two independent tumors from one diseased breast both of which were IDC specimens. From these specimens, total RNA was collected and *DSC3 *expression was analyzed by quantitative real time RT-PCR with expression levels of the tumor samples being normalized to HMECs. DSCs are expressed in both 'a' and 'b' isoforms as a result of alternate splicing of exon 16. The ABI probe used in these studies spans exon1 and exon2, which are both conserved in the *DSC3a *and *DSC3b *isoforms, allowing us to analyze both isoforms in the specimens tested. *DSC3 *expression is reduced to less than 10% of the expression seen in HMEC in 18 of 24 (75%) of the IDCs, 5 of 7 (71%) ILCs, and in the mucinous carcinoma (Table 1). The 10% cutoff was chosen to address the potential of reduced expression of *DSC3 *due to contaminating stromal, non-epithelial elements. The majority of specimens analyzed consisted of 50% tumor based on pathology examination. Thus, the greatly reduced expression of *DSC3 *is a common event in primary breast tumor specimens.

**Figure 2:**
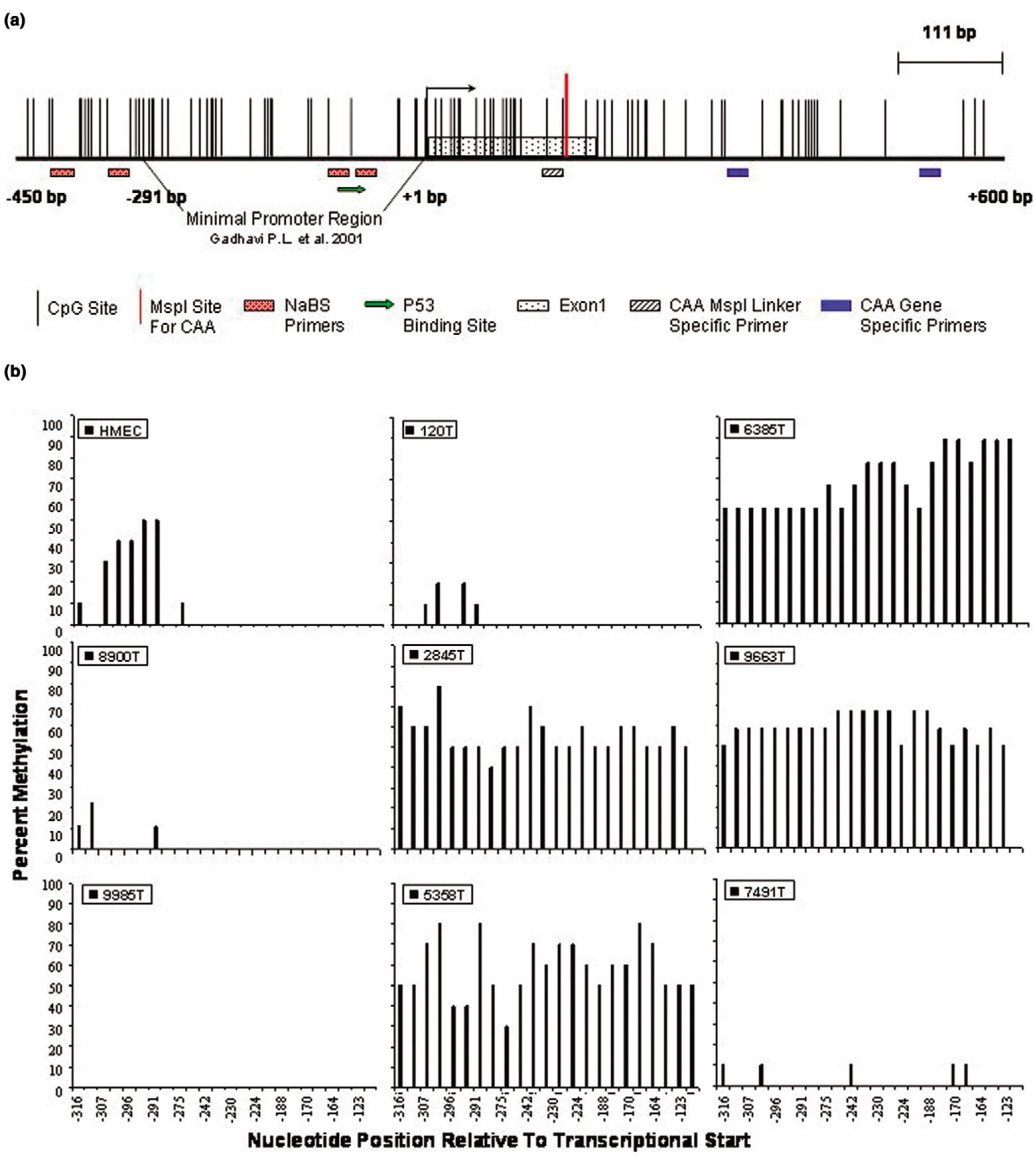
The *DSC3 *promoter is aberrantly methylated in primary breast tumor samples. **(a) **Diagram of the *DSC3 *promoter region analyzed (with the minimal promoter region demarcated as described in [36]). **(b) **Summary of 5-methylcytosine levels obtained by sodium bisulfite genomic sequencing of the *DSC3 *promoter. Ten to twelve cloned PCR products were sequenced to determine the percent methylation of the 24 CpG sites in the region analyzed. Cytosine methylation frequency histograms are shown for normal HMECs and eight primary tumor specimens. The y-axis is percent cytosine methylation and the x-axis is the nucleotide position relative to the transcription start site.

We next examined the cytosine methylation profiles of the breast tumor specimens to see if loss of expression correlates with cytosine methylation of the promoter region (Table 1, Fig. 2b). The *DSC3 *promoter region meets the criteria of a CpG island based on size, GC content, CpG dinucleotide frequency, as well as its location with respect to the transcriptional unit (Fig. 2a). We used sodium bisulfite genomic sequencing to assess the cytosine methylation status of 24 CpG dinucleotides within the *DSC3 *promoter region upstream of the *DSC3 *transcriptional start site. The region analyzed consists of the p53 binding site, the minimal promoter region, and 75 to 100 bases immediately 5' of the minimal promoter region [6,36]. Ten to twelve cloned PCR products were sequenced to determine the percent methylation of the 24 CpG sites in the 5' promoter region. Of the 18 IDC samples that showed a loss of *DSC3 *expression, 10 (56%) of these specimens contained methylated cytosines within the CpG island. In the eight remaining IDC specimens that lack *DSC3 *expression we predict that other mechanisms of silencing such as mutation to p53 or loss of other transcription factors are participating in *DSC3 *gene silencing. Of the five ILC specimens that lacked *DSC3*, two (40%) were shown to contain methylated CpG islands. The one mucinous carcinoma specimen analyzed showed a loss of *DSC3 *expression with a concomitant increase in cytosine methylation. In addition, we analyzed two benign fibrocystic disease specimens and in both cases we saw no methylation of the CpG island and *DSC3 *gene expression in one of two specimens analyzed. At the very 5' region we saw CpG sites that show methylation variable positions in many of the *DSC*-positive specimens; we interpret these CpGs to likely be demarcating the edge of the CpG island. Indeed, the first four 5' sites are outside of the minimal promoter region and are likely to be at the edge of the functional CpG island where methylation is more variable [37-39]. Nonetheless, methylation of the *DSC3 *promoter correlates with a lack of expression of *DSC3 *in a significant proportion of the primary tumor specimens examined.

**Figure 3:**
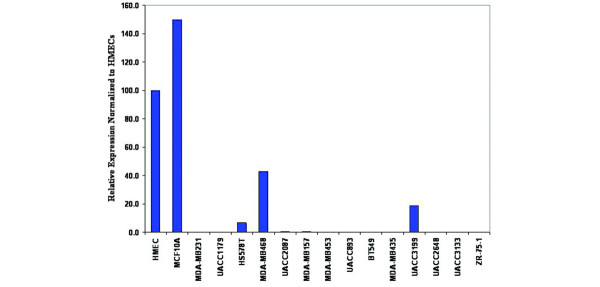
*DSC3 *gene expression is silenced or greatly reduced in a high percentage of breast tumor cell lines. *DSC3 *expression relative to human mammary epithelium cells (HMECs) was assessed by real-time quantitative RT-PCR; *GAPDH *expression was used to normalize the data.

To further characterize *in vitro *models for studying the epigenetic state of the *DSC3 *promoter we extended prior studies [6,29] and analyzed 14 human breast tumor cell lines for *DSC3 *expression by quantitative real time RT-PCR. Tumor expression levels were normalized to *GAPDH*, and expression was then compared to HMECs. Normalized expression levels are shown in Fig. 3. In the breast tumor cell lines tested, 11 of 14 (79%) showed a loss of *DSC3 *expression, whereas HS578T, MDA-MB-468, and UACC3199 showed moderate expression levels. Of note, three of the breast tumor cell lines tested, BT549, MDA-MB-231, and MDA-MB-157, are in agreement with earlier findings [29].

**Figure 4:**
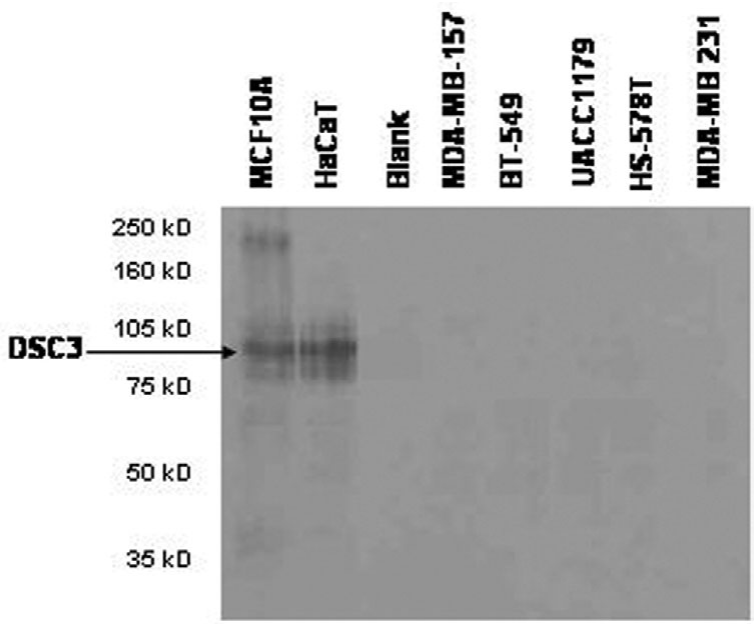
DSC3 protein is not expressed in breast tumor cells with undetectable *DSC3 *mRNA levels. Protein expression was analyzed by western blot analysis. MCF10A and HaCaT cells were used as positive controls for DSC3 expression.

To determine if loss of mRNA expression correlated with a decrease in protein levels, we conducted western blot analysis of DSC3 in a select group of cell lines. Chosen for analysis were the MDA-MB-157, MDA-MB-231, UACC1179, HS578T, and BT549 breast tumor cell lines, as well as the immortalized but non-tumorigenic breast epithelial cell line MCF10A. HaCaT cells, which are a spontaneously immortalized human keratinocyte cell line, served as a positive control for DSC3 expression [40]. The lack of mRNA expression resulted in a marked reduction of DSC3 protein expression in the cell lines tested (Fig. 4). The HS578T cell line, which showed a 7% expression of *DSC3 *mRNA, did not produce any detectable protein expression, which is likely below the limit of detection for the western blot conducted. MCF10A cells, which express *DSC3 *mRNA, showed protein expression comparable to the HaCaT cells; however, no protein bands were present in any of the tumor cell lines examined. Therefore, the lack of *DSC3 *mRNA expression results in a significant loss of DSC3 protein expression in breast tumor cell lines.

**Figure 5:**
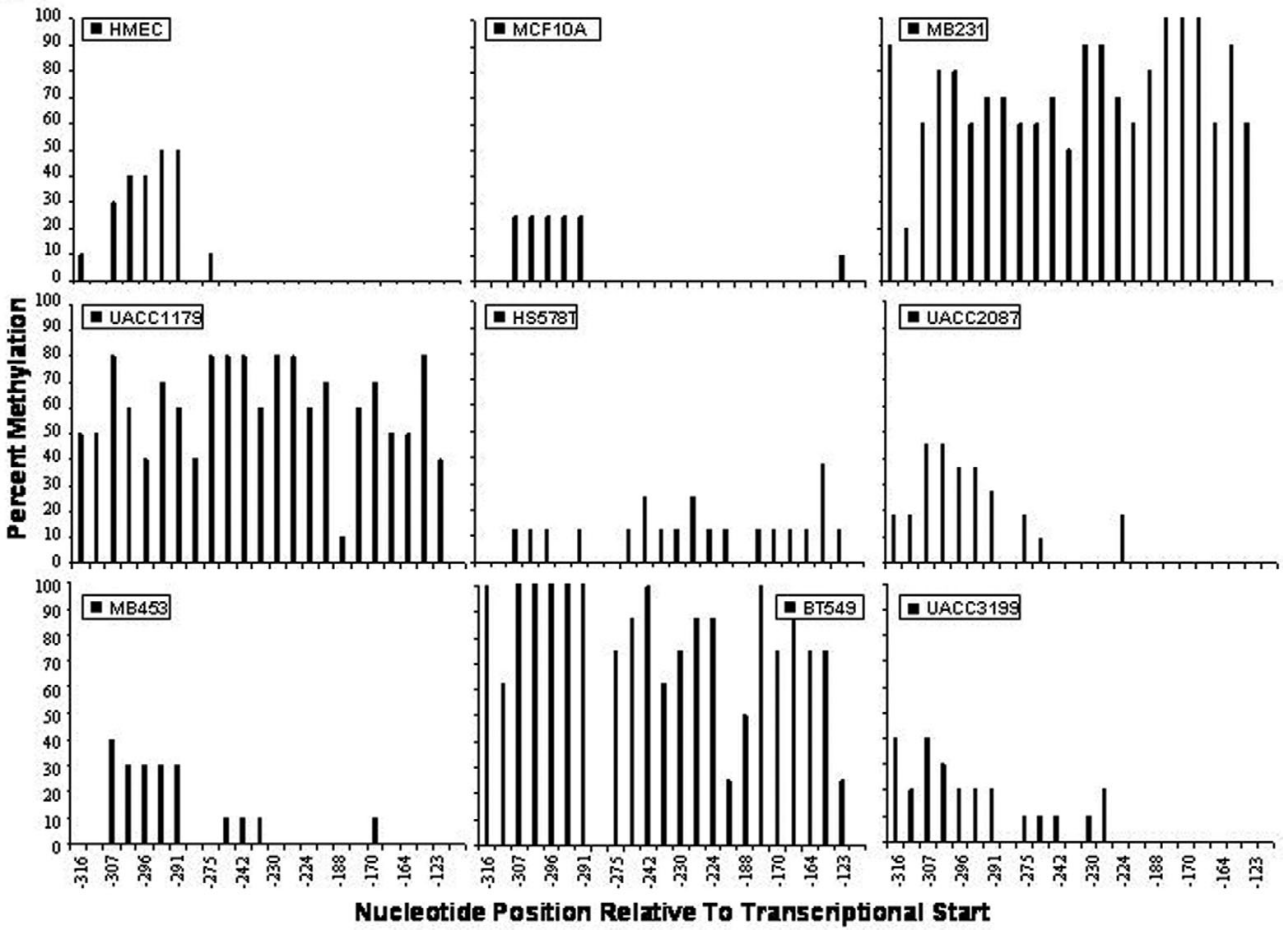
The *DSC3 *promoter is aberrantly methylated in breast tumor cell lines. Ten to twelve cloned PCR products were sequenced to determine the percent methylation of the 24 CpG sites in the region analyzed. Cytosine methylation frequency histograms are shown for human mammary epithelium cells (HMECs) and the immortalized non-tumorigenic MCF10A cells, and seven human breast cancer cell lines examined. The y-axis is percent cytosine methylation and the x-axis is the nucleotide position relative to the transcription start site.

To determine if *DSC3 *expression is lost in association with aberrant methylation of the *DSC3 *promoter we used sodium bisulfite genomic sequencing to assess the cytosine methylation status of the *DSC3 *promoter region. Again, 10 to 12 cloned PCR products were sequenced to determine the percent methylation of the 24 CpG sites in the 5' promoter region. The DSC3 promoter region was relatively unmethylated in the *DSC3*-positive, HMECs, and MCF10A cells (Fig. 5). In the *DSC3*-negative cell lines MB231, UACC1179, and BT549, there is a strong correlation between cytosine methylation of the promoter region and lack of expression. Interestingly, in the two remaining *DSC3*-negative cell lines, UACC2087 and MB-453, loss of expression does not correlate with cytosine methylation of its promoter region, which suggests that other mechanisms of gene silencing are present in these cell lines. These results are similar to the conditions found in the clinical specimens where *DSC3 *is silenced due to cytosine methylation of its promoter region in 41% of specimens analyzed. Notably, as each of these cell lines contain mutant p53, the loss of this transcription factor is likely participating in the silencing of *DSC3 *[6,41]. Finally, in the two remaining tumor cell lines that express *DSC3 *we saw little or no methylation of the promoter region. Therefore, the lack of *DSC3 *expression in these breast tumor specimens is due in part to both epigenetic and genetic mechanisms of gene silencing.

**Figure 6:**
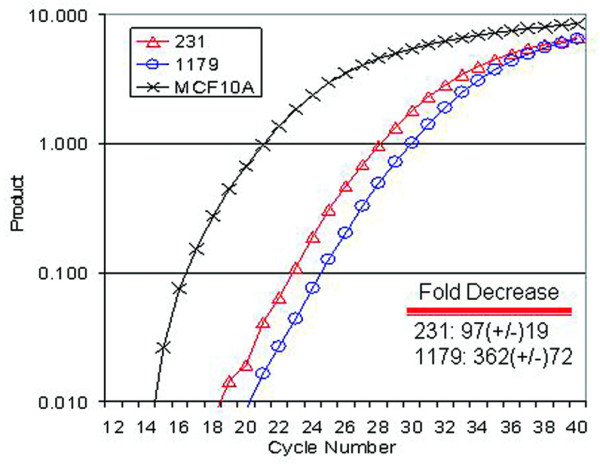
Hypermethylated *DSC3 *promoter regions are inaccessible to *in vivo *MspI endonuclease digestion. Intact nuclei were isolated from MDA-MB-231 and UACC1179 cells and digested *in vivo *with MspI. Isolated DNA was ligated with a linker specific to the MspI ends, and hemi-nested, linker mediated PCR was conducted with two rounds of PCR with two gene specific primers. Increased amounts of PCR product reveal the presence of accessible chromatin. Inset within the graph is the average calculated fold decrease and standard deviation when MDA-MB-231 and UACC1179 cells are compared to MCF10A. The graph shown is representative of three independent replicates.

Another facet of epigenetic regulation causally linked to aberrant cytosine methylation is localized changes to chromatin architecture. Generally, methylated and silenced regions are associated with a 'closed' chromatin structure whereas unmethylated and transcriptionally competent regions are associated with an 'open' chromatin structure. We therefore analyzed the chromatin structure of the *DSC3 *CpG island region by measuring the accessibility of MspI to its cognate binding site (CCGG) (Fig. 2a) using a quantitative real-time, linker-mediated PCR approach [6]. The PCR for this assay involved a hemi-nested amplification approach, with one primer being specific to the ligated linker and two gene specific primers. The first round of PCR used the linker specific primer and a downstream gene specific primer, while the second round used a portion of the first round product and a gene specific primer 3' to that of the first round gene specific primer to increase specificity of the reaction. Using this technique, we showed a 6.5 to 8.5 cycle difference, which translates to a 90 to 362-fold decrease in chromatin accessibility between the two tumor cell lines tested in comparison to MCF10A cells (Fig. 6). Therefore, *DSC3 *gene silencing is linked to aberrant cytosine methylation and a closed chromatin structure.

## Discussion

The purpose of this study was to determine the frequency of *DSC3 *gene silencing in primary breast tumor specimens and to determine if the loss of expression was due to the aberrant methylation of the *DSC3 *CpG island promoter. Analysis of a panel of normal tissue revealed *DSC3 *mRNA expression to be limited to cell types of epithelial origin. The loss of *DSC3 *expression in primary breast carcinomas and tumor cell lines has been previously reported [29]. We extended these studies and show that downregulation of *DSC3 *is a common event in both primary breast tumors as well as breast tumor cell lines, in which we saw a 72% and 79% loss of expression, respectively. *DSC3 *gene silencing is linked to aberrant cytosine methylation in 41% of the primary breast carcinomas tested. Furthermore, using *in vitro *models we show that the epigenetic silencing of *DSC3 *is due in part to cytosine methylation of its promoter region and to concomitant changes in chromatin structure that lead to it forming a closed, inaccessible conformation in the breast cancer cell lines.

We analyzed the cytosine methylation status of 24 CpG sites just 5' of transcriptional start. Within the first seven CpG sites analyzed, we saw methylation variable positions in nearly all primary tumors and cell lines analyzed. This finding signifies to us that the first seven sites are at the very edge of the functional CpG island, where methylation tends to be more variable and coincidentally resides outside of the defined minimal promoter [36-39]. The last 17 CpGs analyzed are within the minimal human promoter region previously identified and are almost completely unmethylated in *DSC3*-positive cells. Therefore, cytosine methylation within the DSC3 minimal promoter region results in the silencing of *DSC3 *gene expression.

Of the primary tumor specimens exhibiting a loss of *DSC3 *expression, several were not associated with cytosine methylation of its promoter region. In these particular cases we predict that the loss of critical transcription factors may be contributing to the silencing of gene expression. Notably, we have shown [6] that *DSC3 *is a p53 response gene and that the addition of wild-type p53 is sufficient to induce acetylation of the *DSC3 *promoter region and induce re-expression of *DSC3 *in breast tumors. Thus, we hypothesize that loss of transcription factors is an early event in tumor progression. We further hypothesize that subsequent to the loss of critical transcription factors, the promoter regions become 'unprotected' and aberrant cytosine methylation that occurs in the region induces long term gene silencing, similar to epigenetically regulated cell type-specific genes [32].

While functional studies have identified *DSC3 *as a potential tumor suppressor gene [27], future studies are necessary to determine the role of DSC3 in breast tumor initiation and progression. Compelling evidence in the literature indicates an important role for the loss of cellular adhesion in breast tumor progression. In an elegant study, Sternlicht *et al*. [42] showed that transgenic mice that express an auto-activating form of MMP-3/stromelysin-1, under the control of the whey acidic protein gene promoter, undergo spontaneous development of premalignant and malignant lesions in the mammary glands when compared to their non-transgenic littermates. This study, conducted over a two year period, shows that the single addition of *MMP-3*, a gene that encodes an enzyme that degrades extracellular components such as fibronectin, laminin, collagens III, IV, IX, and X, and cartilage proteoglycans, is sufficient to induce moderate to severe mammary hyperplasia, lymphocytic infiltrates, ductal carcinoma *in situ*, and mammary carcinomas. The loss of cell adhesion molecules is thus sufficient to induce neoplastic mammary diseases and warrants the further investigation of the role of desmosomal protein loss in breast tumor progression. Furthermore, desmosomal proteins such as DSC3 have been shown to be critical for desmosome formation, cell position, and inhibition of cell motility [27,43]. As such, the identification of *DSC3 *as a gene that is commonly downregulated in breast cancer necessitates the need for further examination of its role in breast tumor progression.

## Conclusion

The finding that the *DSC3 *gene is frequently silenced by epigenetic mechanisms in breast cancer opens new avenues to understanding the underlying causes of malignant progression in breast cancer and helps to identify new targets for therapeutic intervention.

## Abbreviations

bp = base pair; DSC = desmocollin; H&E = hematoxylin and eosin; HIPAA = health insurance portability and accountability act of 1996; HMEC = human mammary epithelial cell; IDC = invasive ductal carcinoma; ILC = invasive lobular carcinoma; PBS = phosphate-buffered saline.

## Competing interests

The authors declare that they have no competing interests.

## Authors' contributions

MMO helped develop the methods to isolate RNA and DNA from patient specimens, participated in the sodium bisulfite sequencing analysis, helped in the design of the study, and drafted the manuscript. CJK is the collaborating surgical oncologist responsible for obtaining patient specimens in accordance with HIPAA guidelines and also participated in the pathology review of all specimens. RJW helped develop and implement the chromatin accessibility assay for this study. DJJ conducted the microarray study that identified DSC3. JLMR developed the primers for use in the sodium bisulfite sequence analysis and also participated in the sequencing of the patient specimens. JAB was responsible for RNA and DNA isolations from all patient specimens, conducted the real-time RT-PCR analysis of patient specimens, and also participated in sodium bisulfite sequence analysis. MF participated in sodium bisulfite sequence analysis. SP conducted the protein isolations and performed the western blot analysis. AEC, an expert in cell adhesion, helped to critically review the manuscript and provided the resources for the western blot analysis. FED, an expert in the field of epigenetics, contributed to the design of the study, participated in and provided the resources for sodium bisulfite sequencing of DSC3, and critically reviewed the manuscript. BWF conceived of the study, participated in its design and coordination and helped to draft the manuscript. All authors read and approved the final manuscript

## Figures and Tables

**Table 1:** Summary of *DSC3 *expression and methylation state in primary breast tumors

Cell/tumor	Expression^a^	Methylation^b^	%Me^c^	Age (years)	Histology
HMEC	100.0%	-	15	N/A	N/A
MCF10A	150.0%	-	7	N/A	N/A
120T	81.4%	-	3	59	IDC
7732T	6.9%	-	10	44	IDC
6385T	0.0%	++	69	70	IDC
173T	0.1%	+	30	55	IDC
8900T	77.2%	-	2	56	IDC
4658T	52.4%	-	8	83	IDC
7768T	2.3%	-	7	40	IDC
2504T	67.8%	-	16	53	IDC
9613T	8.0%	-	9	42	IDC
6010T	0.3%	+	21	55	IDC
2909T	26.3%	-	1	38	IDC
2845T	0.0%	++	49	77	IDC
^d^5974-1T	10.0%	+	23	43	IDC
^d^5974-2T	10.0%	++	48	43	IDC
7093T	10.0%	+	30	63	IDC
9068T	0.0%	-	8	73	IDC
4392T	0.0%	+	33	47	IDC
5799T	0.0%	+	22	53	IDC
6245T	0.0%	-	1	30	IDC
2405T	30.0%	+	20	58	IDC
2420T	0.0%	-	8	54	IDC
5256T	3.9%	-	1	60	IDC
1139T	6.4%	-	11	40	IDC lymph node Met.
9663T	0.1%	+	60	41	IDC lymph node Met.
9985T	5.8%	-	0	61	ILC
7788T	1.3%	+	23	76	ILC
6861T	20.2%	+	29	71	ILC
5358T	73.9%	++	58	42	ILC
6608T	5.6%	-	8	74	ILC
7491T	30.0%	-	2	64	ILC
6809T	0.0%	++	49	57	ILC
4099T	0.6%	+	37	43	Mucoid ductal CA

^a^RNA expression levels determined by quantitative real-time PCR and relative to human mammary epithelial cells (HMECs). ^b^CpG island methylation levels determined by bisulfite sequencing: ++, >40% methylation of total CpG sites analyzed; +, >20% methylation of total CpG sites; -, <20% methylation of total CpG sites analyzed. ^c^Percent methylation was calculated based on the number of methylated CpG sites compared to the total number of sites analyzed. ^d^Two independent tumors isolated from the same breast. IDC, invasive ductal carcinoma; ILC, invasive lobular carcinoma; N/A, not applicable, IDC lymph node Met, invasive ductal carcinoma that metastasized to the lymph node, Mucoiod Ductal CA, mucinous ductal carcinoma.
